# A Systematic Review and Meta-Analysis of the In Vivo Haemodynamic Effects of Δ^9^-Tetrahydrocannabinol

**DOI:** 10.3390/ph11010013

**Published:** 2018-01-31

**Authors:** Salahaden R. Sultan, Sophie A. Millar, Saoirse E. O’Sullivan, Timothy J. England

**Affiliations:** 1 Division of Medical Sciences & Graduate Entry Medicine, School of Medicine, University of Nottingham, Derby DE22 3DT, UK; mzxss4@nottingham.ac.uk (S.R.S.); stxsamil@nottingham.ac.uk (S.A.M.); mbzso@nottingham.ac.uk (S.E.O.); 2 Faculty of Applied Medical Sciences, King Abdulaziz University, Jeddah 21589, Saudi Arabia

**Keywords:** ∆^9^-Tetrahydrocannabinol, THC, cardiovascular system, blood pressure, heart rate, blood flow

## Abstract

∆^9^-Tetrahydrocannabinol (THC) has complex effects on the cardiovascular system. We aimed to systematically review studies of THC and haemodynamic alterations. PubMed, Medline, and EMBASE were searched for relevant studies. Changes in blood pressure (BP), heart rate (HR), and blood flow (BF) were analysed using the Cochrane Review Manager Software. Thirty-one studies met the eligibility criteria. Fourteen publications assessed BP (number, *n *= 541), 22 HR (*n *= 567), and 3 BF (*n *= 45). Acute THC dosing reduced BP and HR in anaesthetised animals (BP, mean difference (MD) −19.7 mmHg, *p *< 0.00001; HR, MD −53.49 bpm, *p *< 0.00001), conscious animals (BP, MD −12.3 mmHg, *p *= 0.0007; HR, MD −30.05 bpm, *p *< 0.00001), and animal models of stress or hypertension (BP, MD −61.37 mmHg, *p *= 0.03) and increased cerebral BF in murine stroke models (MD 32.35%, *p *< 0.00001). Chronic dosing increased BF in large arteries in anaesthetised animals (MD 21.95 mL/min, *p *= 0.05) and reduced BP in models of stress or hypertension (MD −22.09 mmHg, *p *< 0.00001). In humans, acute administration increased HR (MD 8.16 bpm, *p *< 0.00001). THC acts differently according to species and experimental conditions, causing bradycardia, hypotension and increased BF in animals; and causing increased HR in humans. Data is limited, and further studies assessing THC-induced haemodynamic changes in humans should be considered.

## 1. Introduction

∆^9^-Tetrahydrocannabinol (THC) is the most abundant and widely studied phytocannabinoid, first discovered in 1964 [1]. THC is a partial agonist of both cannabinoid receptors CB_1_ and CB_2_ and other targets including G protein-coupled receptors GPR55 and GPR18 [2,3,4]. THC possesses interesting therapeutic potential as an antiemetic, appetite stimulant, and analgesic, and for the treatment of glaucoma, epilepsy, Parkinson`s disease, and multiple sclerosis [5,6,7]. THC has been shown to be effective against refractory nausea and vomiting in cancer patients undergoing chemotherapy [8]. However, its use as a therapeutic agent is limited by its recognised psychogenic side effects including hallucinations, euphoria, dizziness, mood changes, nausea, and fatigue [8,9,10].

THC has numerous cardiovascular effects in animals and humans. In vitro studies have shown that THC causes endothelium-independent vasorelaxation of rabbit superior mesenteric arteries [11] and vasorelaxation of the rat mesenteric artery through sensory nerves via a CB_1_ and CB_2_ receptor-independent mechanism [12]. Other studies have found THC to activate a G protein-coupled receptor, inhibit calcium channels, and activate potassium channels in the rat mesenteric vasculature [13] and to cause endothelium-dependent and time-dependent vasorelaxation in the rat aorta [14,15]. In contrast, other studies have shown that THC causes vasoconstriction in guinea pig pulmonary arteries [16], rat mesenteric arteries and aorta [14,17], and rabbit ear arteries [18].

In vivo studies have reported different haemodynamic responses post-THC. An acute administration of THC caused hypotension and bradycardia in anesthetised dogs (intravenously; i.v.), conscious bats (intraperitoneal; i.p.), and humans (oral) [19,20,21]. In contrast, tachycardia and hypertension were reported in rats after i.p. administration of THC [22,23]. More complex effects on BP were induced by THC in anaesthetised rats [24]. The available evidence to date suggests that THC alters the haemodynamics in animals and humans, albeit with conflicting results variable with species, route of administration, and experimental conditions. Therefore, the aim this study was to systematically review and meta-analyse the in vivo literature assessing the effects of THC on the cardiovascular system in all species under different conditions.

## 2. Results

**Figure 1:**
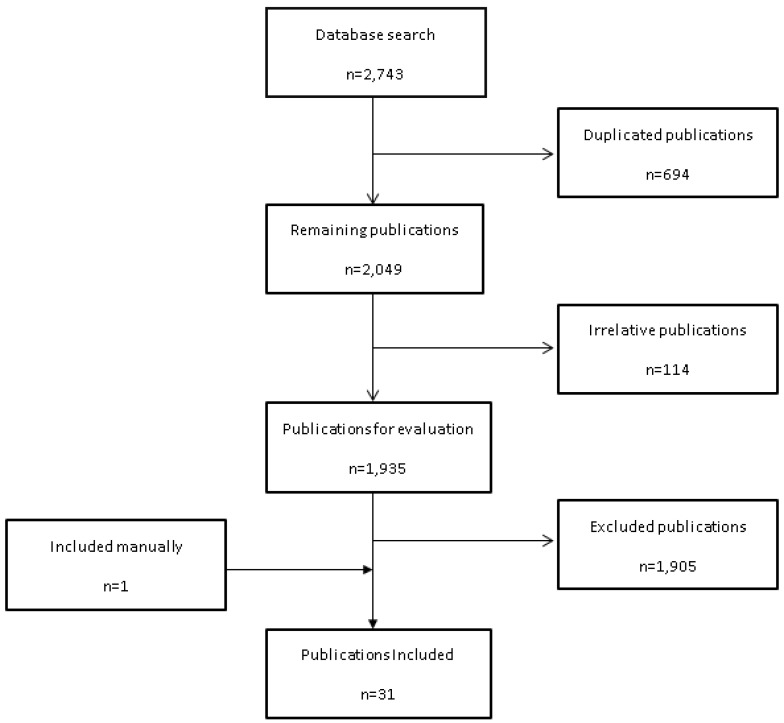
Flow chart for study retrieval and selection.

From the initial 2743 search results, 1935 relevant publications were identified and evaluated from three databases (Medline, EMBASE, and PubMed). Of these, 30 articles met the inclusion criteria and 1 article was added manually (Figure 1). A summary of the data extracted from the included studies is shown in Table 1.

### 2.1. Effect of THC Treatment on Haemodynamics

#### 2.1.1. Anaesthetised Animals

**Figure 2:**
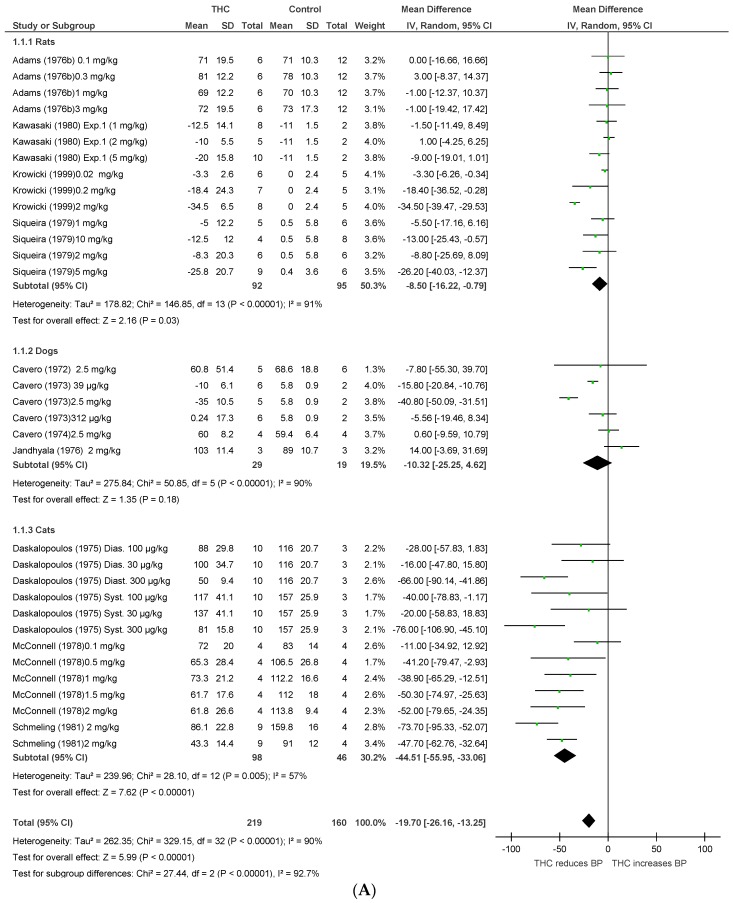

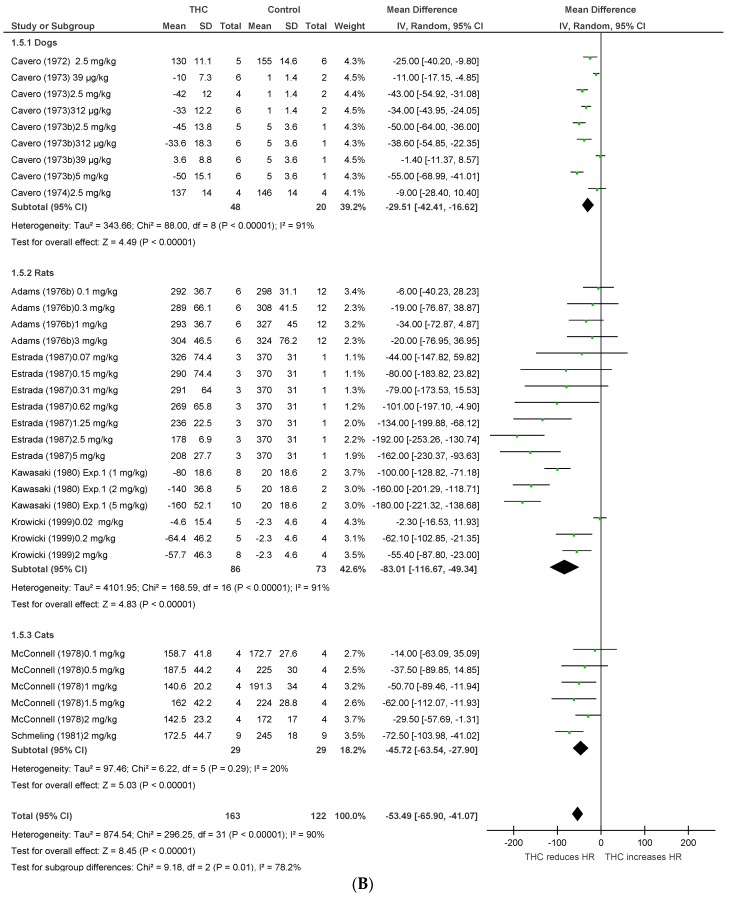
Changes in (**A**) BP and (**B**) HR induced by acute THC dosing in anaesthetised animals.

Fifteen publications [19,23,24,25,26,27,28,29,30,31,32,33,34,35,36] assessed the effect of THC administration in three anaesthetised species (rats, dogs, and cats, *n* = 664). THC significantly reduced BP and HR after acute dosing (BP, MD −19.7 mmHg, 95%CI −26.16, −13.25, *p* < 0.00001; HR, MD −53.49 bpm, 95%CI −65.9, −41.07, *p* < 0.00001, Figure 2A,B). A cross-species analysis revealed that THC responses in the three species were significantly different in both BP (*p* < 0.00001) and HR (*p* = 0.01) (Figure 2A,B), and acute THC significantly reduced BP in rats and cats, but not in anesthetised dogs (*p* = 0.18, Figure 2A).

**Figure 3:**
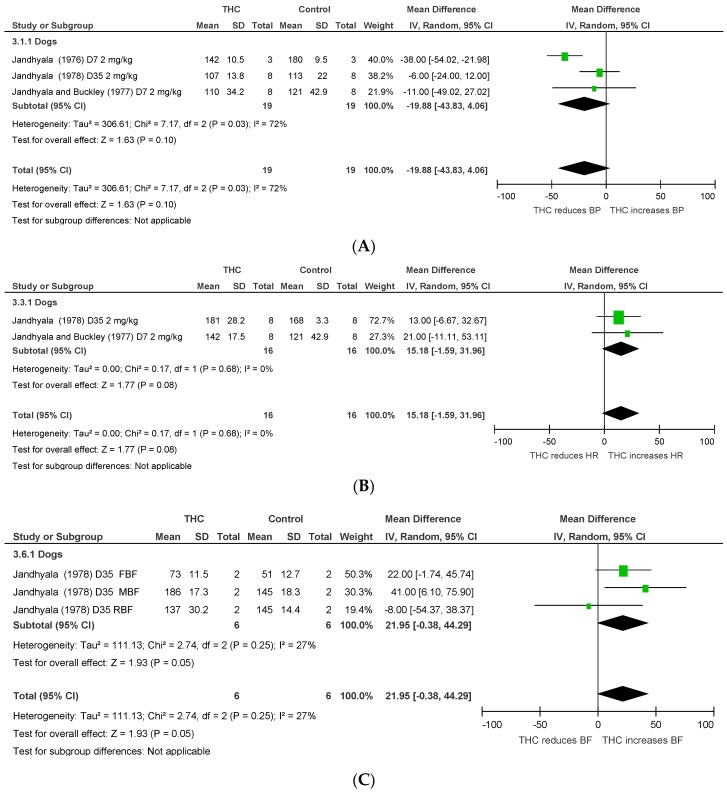
Changes in (**A**) blood pressure, (**B**) heart rate, and (**C**) blood flow (BF) induced by chronic THC dosing in anaesthetised animals.

Chronic THC administration (7–35 days) tended to increase mesenteric, femoral, and renal BF (*p* = 0.05, Figure 3C) with no significant effect on HR or BP. Heterogeneity was statistically significant for BP and HR measurements after acute THC dosing (*p* < 0.00001; I^2^ = 90%) and for BP after chronic THC dosing (BP, *p* = 0.03, I^2^ = 72%).

#### 2.1.2. Conscious Animals

**Figure 4:**
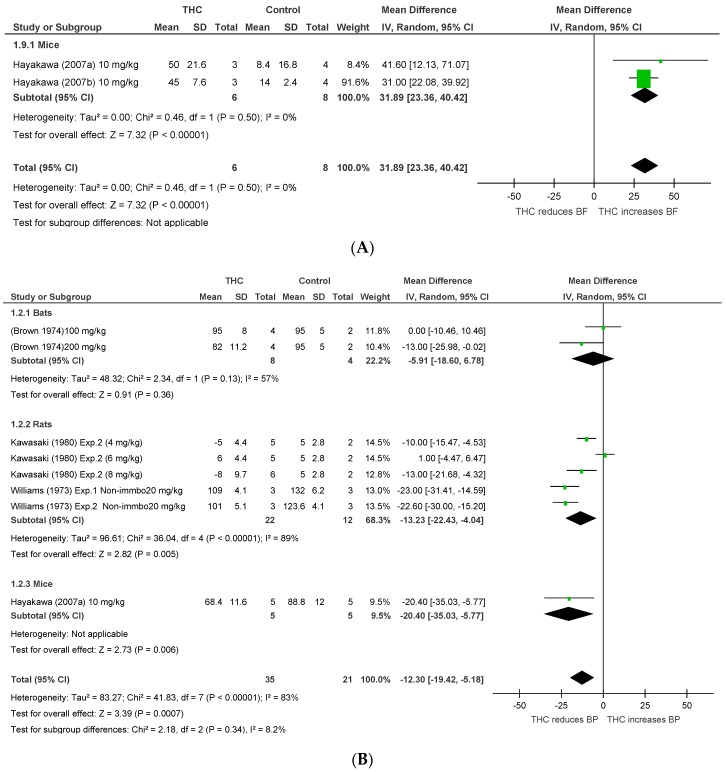

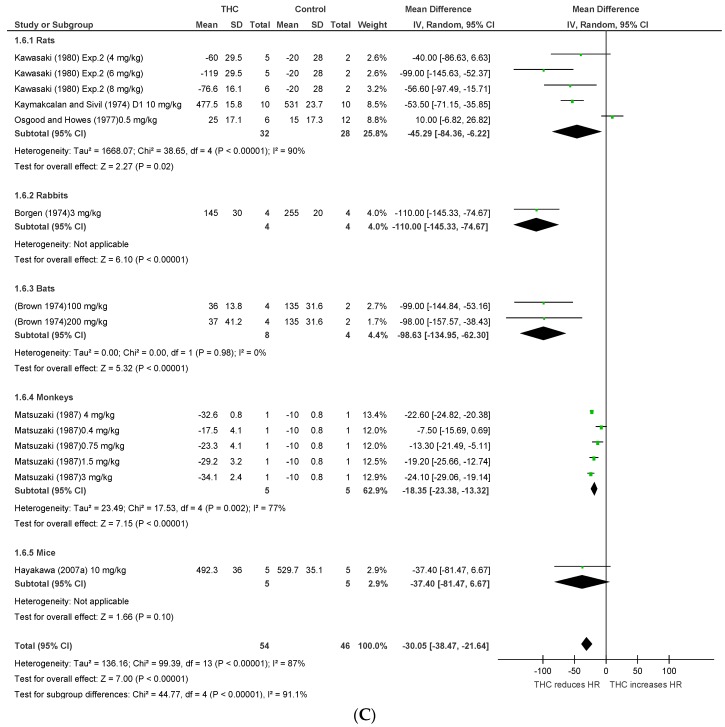
Changes in (**A**) BP, (**B**) HR, and (**C**) blood flow induced by acute THC dosing in conscious animals.

Eight publications [20,22,23,37,38,39,40,41] assessed the effect of THC administration in five conscious species, including rats, bats, mice, rabbits, and monkeys (*n* = 170). THC significantly reduced BP and HR after acute dosing (BP, MD −12.3 mmHg, 95%CI −19.42, −5.18, *p* = 0.0007; HR, MD −30.05 bpm, 95%CI −38.47, −21.64, *p* < 0.00001, Figure 4A,B), and significantly increased CBF in murine models of stroke (BF, MD 32.35%, 95%CI 23.81, 40.88, *p* < 0.00001, Figure 4C). A cross-species analysis revealed that acute THC did not affect BP in bats (*p* = 0.36) and rats (*p* = 0.11) (Figure 4B). Heterogeneity was statistically significant for BP and HR measurements after acute THC dosing (BP, *p* < 0.00001, I^2^ = 83%; HR, *p* < 0.00001, I^2^ = 87%), but not in BF (*p* = 0.5, I^2^ = 0%).

#### 2.1.3. Conscious Animal Models of Stress or Hypertension

**Figure 5:**
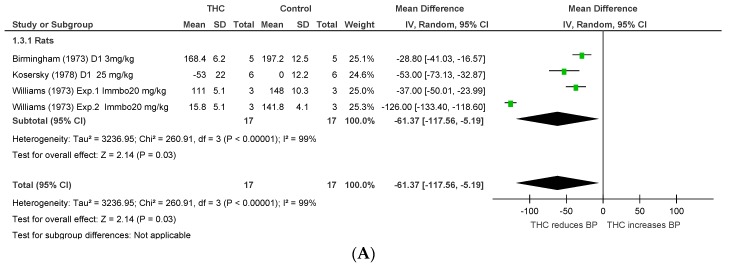

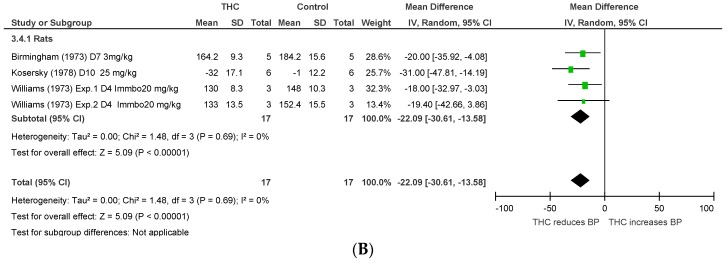
Changes in BP induced by (**A**) acute and (**B**) chronic THC dosing in animal models of stress or hypertension.

Two publications [43,44] assessed the effect of THC administration on BP in hypertensive rats (*n* = 22), and one [42] in a rat model of stress (*n* = 30). Acute and chronic (4–10 days) THC dosing significantly reduced BP (acute THC, MD −61.37 mmHg, 95% CI –117.56, −5.17, *p* = 0.03, Figure 5A; chronic THC, MD −22.09 mmHg, 95% CI −30.61, −13.58, *p* < 0.00001, Figure 5B). Heterogeneity was statistically significant after acute dosing (*p* < 0.00001, I^2^ = 99%), but not after chronic dosing (*p* = 0.69, I^2^ = 0%).

#### 2.1.4. Human Studies

**Figure 6:**
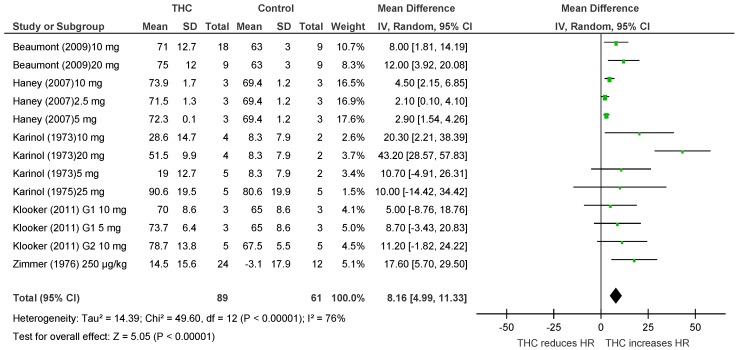
Changes in HR induced by acute THC dosing in humans.

Six publications [21,45,46,47,48,49] assessed the acute effect of THC administration on HR in humans (*n* = 150), no studies examined BP or BF. THC significantly increased HR after acute dosing (HR, MD 8.16 bpm, 95% CI 4.99, 11.33, *p* < 0.00001, Figure 6). Heterogeneity was statistically significant (*p* < 0.00001; I^2^ = 76%).

### 2.2. Dose–Response to THC

**Figure 7:**
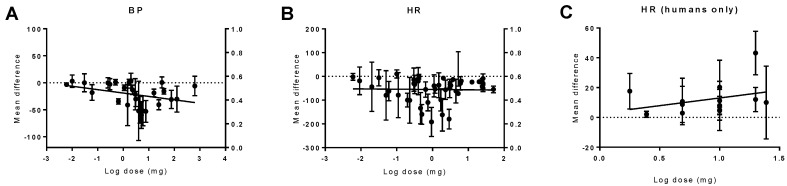
The effect of different THC doses on haemodynamic responses in vivo. The mean difference (MD) in animals’ blood pressure (BP, (**A**)), animals’ heart rate (HR, (**B**)), or heart rate (in humans only) (*p* = 0.01) (HR, (**C**)) is plotted against the log dose (mg) for each study. Error bars represent 95% confidence intervals (CI). Near-significant and significant dose-dependent effects on the blood pressure in animals (*p* = 0.07) and on the HR in humans (*p* = 0.01).

Doses ranging from 0.0003 to 770 mg were used in different species. The animal analyses showed a trend in the reduction of BP with higher THC doses (*p* = 0.07), with no change in HR. In humans, THC caused dose-dependent tachycardia (*p* = 0.01) (Figure 7).

### 2.3. Quality

Among the 31 included publications, 6 publications used randomisation in their design and reported blinding assessment of outcome and measurements. Twenty publications assessed more than one outcome, 19 conducted dose–response relationships, 26 assessed a time window for intervention, 11 measured outcomes >24 h post-drug, and no publications provided incomplete data. There was no significant relationship between the quality score and any outcome (Spearman’s rho coefficient of BP 0.22, *p* = 0.09; HR 0.27, *p* = 0.07 and BF 0.58, *p* = 0.3).

### 2.4. Publication Bias

**Figure A1:**
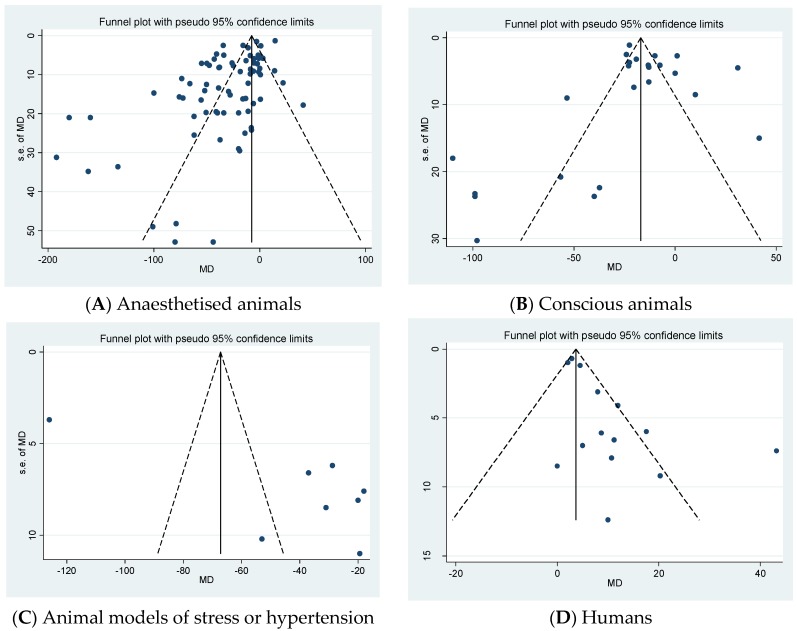
Funnel plots for each outcome evaluating the publication bias. The standard error (SE) of the mean difference (MD) in haemodynamics (MD, *y* axis) for each study is plotted against its effect size (*horizontal* axis). There was significant bias in conscious animals (**B**) (*p* = 0.001), animal models of stress or hypertension (**C**) (*p* = 0.001), and humans (**D**) (*p* < 0.0001). No significant bias in anaesthetised animals (**A**).

Egger’s test showed that bias was present in all studies except in studies in anaesthetised animals, conscious animals (*p* = 0.001), animal models of stress or hypertension (C) (*p* = 0.001), and humans (D) (*p* < 0.0001) (Appendix A, Figure A1).

## 3. Discussion

The aim of this study was to determine the effect of THC on haemodynamics in vivo in animals and cannabis-naïve humans. Our analysis has shown that an acute dosing of THC reduced BP and HR, and increased BF in animals of different models. Chronic dosing of THC tended to increase BF in anaesthetised animals and reduced BP in animal models of stress or hypertension. The data concerning the effects of THC in humans was limited to HR only, revealing a dose-dependent increase, suggesting further work is required to determine the full haemodynamic effects of acute and chronic THC administration in humans, especially given the different effects of THC on HR observed across species.

Our meta-analysis showed that acute THC dosing in anaesthetised animals reduced BP and HR, while a subgroup analysis revealed that there was no effect on BP or HR of anaesthetised dogs. However, Cavero et al. (1972, 1973, 1974) reported that intravenous administration of THC induced hypotension and bradycardia in dogs anaesthetised with pentobarbital caused by a reduction in the cardiac output and venous return mediated by the autonomic system [19,25,26,27]. Similarly, Schmeling reported that the reduction in sympathetic activity induced by THC in cats may cause hypotension and bradycardia [34]. It is suggested that the vagus nerve and the sympathetic outflow play a role in these effects induced by THC [36] and can be inhibited by the administration of a CB_1_ antagonist [50]. The administration of THC for seven days subcutaneously reduced the increase in HR induced by pentobarbital anaesthetic agent in dogs, suggesting that THC antagonises the pentobarbital effect on the parasympathetic system (inhibiting the vagal tone) [30]. In rats anesthetised with pentobarbital, hypotension was reported after the administration of THC [35]; on the contrary, hypertension was reported in rats anesthetised with urethane post-THC [36], suggesting that THC may act differently with different anaesthetic agents. These studies suggest that the effects of THC in anaesthetised animals (hypotension and bradycardia) are induced through a central mechanism via the activation of CB_1_ receptors.

In conscious animals under normal conditions, THC caused a variety of effects: hypotension was observed in bats, an effect which may be related to a change in venous activity [20], whereas another study in rats reported that THC induced tachycardia and hypertension, which are centrally mediated by increasing the level of adrenaline in the circulation [22]. However, studies in rat models of stress and hypertension, showed that THC lowered BP effectively [42,43,44]. The mechanism of the antihypertensive effect of THC in these models still needs to be studied.

Our meta-analysis in cannabis-naïve humans highlighted the limited number of studies investigating the effect of THC in humans (6 publications, *n* = 123 participants) with insufficient data to meta-analyse BP or regional BF. Studies in cannabis-naïve volunteers showed that the administration of THC orally or by inhalation caused tachycardia [46,47,48,49,51]. Tachycardia is also reported in humans after smoking cannabis [52,53,54] which may indicate that tachycardia induced post-cannabis smoking is caused by THC. The increase in HR caused by THC can be inhibited by CB_1_ antagonism [55], suggesting that CB_1_ activation may play a role in the haemodynamic effect of THC in humans. A greater number of studies investigating the haemodynamic effect of THC and its mechanisms under normal and pathological conditions in humans are required.

Several studies have reported that phytocannabinoids such as cannabidiol (CBD) may alter the effect of THC. For example, Borgen and Davis suggested that CBD may act as a potential antagonist of the THC effect on HR in rabbits and rats [38] and protects against some of the negative effects of THC in humans with potentially opposite effects on regional brain functions [56,57]. The combination of CBD and THC such as in Sativex^®^, a licenced agent for the symptomatic treatment of spasticity in multiple sclerosis, has shown that CBD inhibits the tachycardia effect induced by THC in humans [58].

Dose–response analyses showed a relationship between THC dose and effect size on BP, but not HR, in different animal models, and on human HR. Dose-dependent effects on BP were also observed post-THC in anaesthetized rats [24,36], cats [28], and dogs [26]. A dose of 100 and 200 mg caused a dose-dependent reduction on the BP of conscious bats, but not on HR [20]. HR dose-dependent reduction was reported in anaesthetized dogs [26,27] and conscious monkeys [39]. In human studies, doses between 2.5 and 25 mg were used. A dose-dependent increase in HR was observed in humans after oral THC administration of 5, 10, and 20 mg [21,49]. Over-intoxication has been reported after 20 mg of oral administration of THC in 5 of 21 healthy volunteers [48].

There are a number of limitations to consider in this analysis. First, the principal intention of 10 of the included studies was not to assess the cardiovascular effects of THC administration; therefore, the data extracted through secondary haemodynamic outcomes in this meta-analysis is for hypothesis-generating purposes. Second, the results should be interpreted with caution because of the heterogeneity between studies in terms of THC dose, time, and route of administration; the responses to THC will clearly be dependent upon peak plasma concentration, which are not easily comparable across studies. Indeed, a significant statistical heterogeneity was observed in the majority of the meta-analyses. Third, only 6 out of 31 articles used randomisation and described a masked assessment of outcomes, factors that can influence the reported outcomes. However, we found no significant correlation between study quality and effect size in this review.

In conclusion, this study has summarised the in vivo cardiovascular effects of THC administration. Our analysis demonstrates that THC acts differently according to species, causing tachycardia in humans, and bradycardia, hypotension, and an increase in regional BF in animals under different conditions. THC may be a potential future treatment for cardiovascular disorders, though its use as a single agent will be limited by CB_1_ mediated psychogenic side effects, events that could be counterbalanced with other agents such as CBD. Data from human studies using THC alone is limited to heart rate only, thereby further good quality, randomised, blinded studies investigating the haemodynamic effects of THC in humans should be considered.

## 4. Materials and Methods

### 4.1. Search Strategy

All studies investigating the haemodynamic effects of THC (including BP, HR, and BF) were searched for (until April 2017) in Medline, EMBASE, and PubMed. Search keywords included: ∆^9^-Tetrahydrocannabinol, Tetrahydrocannabinol, THC, Dronabinol, Marinol, Nabilone, Namisol, cardiovascular, blood pressure, systolic, diastolic, hypertension, hypotension, heart rate, tachycardia, bradycardia, blood flow, haemodynamic, vasodilation, vasorelaxation, and vasoconstriction. References from the included studies were also hand-searched.

Prespecified inclusion and exclusion criteria were used to prevent bias; the studies had to be in vivo, assess haemodynamics (BP, HR or BF), be original articles, be controlled studies, and use cannabis-naïve participants. Therefore, the exclusion criteria were: *in vitro* studies, mixtures of ∆^9^-THC with other cannabis extracts, studies investigating the interaction of THC with other drugs or cannabinoids, studies not assessing haemodynamics (BP, HR, or BF), review articles, editorials, and uncontrolled studies.

### 4.2. Data Acquisition

Data on BP (mmHg), HR (beats per minute, bpm), and BF (% change from baseline or mL/min) were extracted from the included papers, and the changes in haemodynamics 2 h post-drug after acute THC dosing were used for the analyses. This time point was selected as the peak plasma time is between 30 min and 4 h after oral administration and it was the most common time point when haemodynamics were measured throughout the articles. If there were no measurements taken at this time point (2 h post-drug), the closest time point to 2 h was used for the analyses. In chronic studies, the measurements taken at the end of the studies were used for the analyses. If the exact number of animals used in each drug group was not available, the lowest number of animals within the range given was used for the experimental group (THC), and the highest number was used for the control group. If a crossover design was used in a study, the total number of humans was distributed equally to the two groups. Articles were excluded if data were not available. Grab application (version 1.5) was used to extract values from the figures given in published articles if no values were stated within the text. If the published articles used multiple groups (e.g., to assess dose-dependent effects) with one control group, then the number of humans or animals per control group was divided into the number of comparison groups. For the dose–response analysis, the total dose of the drug administrated up to the time when the haemodynamics was measured was used.

### 4.3. Quality

Eight-point criteria derived from Stroke Therapy Academic Industry Recommendations (STAIR) [59,60,61] and the Cochrane collaborations tool [62] were used to identify the risk of bias. Each of the following criteria was equal to 1 point: randomisation, blinding of outcome assessment, blinding of personnel and participant, assessment of more than one outcome, dose–response relationship, therapeutic time window, assessment of outcome >24 h, and incomplete outcome data.

### 4.4. Data Analysis

The studies were divided into acute and chronic groups. The data from human and animal studies were analysed separately. The animals were divided into two groups, anaesthetised and conscious, as the autonomic nervous system may respond differently in the two conditions [63], then grouped before the analysis in normal and abnormal (i.e., models of stress or hypertension) models and then subgrouped by species (mice, rats, dogs, etc.). For the THC dose–response analysis, the data were grouped according to the endpoint (BP, HR, or BF), and then subgrouped according to the dose. The data from each group were analysed as forest plots using the Cochrane Review Manager software (Version 5.3. Copenhagen: The Nordic Cochrane Centre, The Cochrane Collaboration, 2014), and as funnel plots using Stata (StataCorp. 2009. Stata Statistical Software: Release 11. College Station, TX, USA). Funnel plot asymmetry (publication bias) was assessed by Egger’s test [64]. Stata was also used for meta-regression that described the relationship between THC dose and effect size. PRISM 7 (GraphPad, Software, La Jolla, CA, USA) was used to produce the figures of dose–response. Since heterogeneity was expected between the study protocols (different species, models, dose, and time) random-effect models were used. The results of continuous data are expressed as mean difference (MD) with 95% confidence intervals (CIs). The studies were weighted by sample size, and statistical significance was set at *p* <0.05.

## Figures and Tables

**Table 1:** Summary of the included studies divided according to the experimental conditions.

Author & Year	Study Description	Species, Model (Anaesthetic & Route)	Sample Size	THC Dose	THC Route	Time of THC Administration	Time of Haemodynamic Measurements	Basal Parameters *	Outcomes and Comments
**Anaesthetised animals**									
Cavero 1972 [25]	Investigate the haemodynamic effects of THC	Dogs Anaesthetised (pentobarbital, iv)	11	2.5 mg/kg	i.v.	Post-anaesthesia	Continues for 30 m post-drug	-	THC altered distribution of regional BF, and reduced HR and BP.
Cavero 1973a [26]	Investigate the haemodynamic effects of THC	Dogs Anaesthetised (pentobarbital, iv)	23	39 µg/kg–2.5 mg/kg	i.v.	Post-anaesthesia	Continues for 2 h post-drug	C: HR:169, BP:91.7; T: HR:165.7, BP:93.5	THC caused reduction in HR and BP mediated via central nervous system.
Cavero 1973b [27]	Characterise the mechanism of action of THC on HR	Dogs Anaesthetised (pentobarbital, iv)	29	39 µg/kg–5 mg/kg	i.v.	Post-anaesthesia	Continues for 140 m post-drug	-	THC induced reduction in HR through alteration of autonomic innervation to myocardium.
Cavero 1974 [19]	Investigate the effect of THC on venous return	Dogs (heart bypass) Anaesthetised (dibucaine, spinal)	8	2.5 mg/kg	i.v.	Post-anaesthesia	Pre-drug and continues for 30 m post-drug	C: HR:156, BP:85.8; T: HR:147, BP:85.	THC caused reduction in HR and BP, and reduced venous return.
Daskalopoulos 1975 [28]	Investigate the mechanism of THC on CV system	Cats Anaesthetised (urethane, iv)	40	30–300 µg/kg	i.v.	Post-anaesthesia	20 m post-drug	-	THC reduced HR and BP mediated via central nervous system.
Adams 1976 [29]	Examined the CV effects of THC	Rats Anaesthetised (urethane, ip)	72	0.1–3 mg/kg	i.v.	Post-anaesthesia	Continues for 30 min post-drug	C: HR:316.2, BP:76.2; T: HR:314.8, BP:73.5.	THC caused reduction in HR and biphasic BP response (↑ BP followed by ↓ BP), suggesting that THC depressed CV reflex functions.
Jandhyala 1976 [30]	Evaluated possible interaction with THC on HR	Dogs Anaesthetised (pentobarbital)	12	1 mg/kg	s.c.	Twice/day for 7 days Pre-anaesthesia	On the 7th day post-anaesthesia	-	Chronic THC antagonised the elevation in HR induced by the anaesthetic agent via vagal stimulation.
Jandhyala 1977 [31]	Determined chronic administration of THC on CV function	Dogs Anaesthetised (pentobarbital)	16	1 mg/kg	s.c.	Twice/day for 7 days Pre-anaesthesia	On the 7th day post-anaesthesia	-	Chronic THC had no effect on haemodynamics.
Jandhyala 1978 [32]	Investigated prolonged THC effects on CV system	Dogs Anaesthetised (pentobarbital)	16	2 mg/kg	s.c.	Single dose per day for 35 days	On the 35th day post-anaesthesia	-	Chronic THC increased BF in femoral and mesenteric arteries with no effect on HR or BP.
McConnell 1978 [33]	Examined the effects of THC on salivary flow	Cats Anaesthetised (urethane & pentobarbital, ip)	20	0.1–2 mg/kg	i.v.	Post-anaesthesia	Continues for 1 h post-drug	-	THC had no effect in stimulated salivary flow of cats. THC caused a reduction in HR and BP.
Siqueira 1979 [24]	Clarify the triple BP response post-THC	Rats Anaesthetised (urethane, ip)	50	1–10 mg/kg	i.v.	Post-anaesthesia	Continues for 70 m post-drug	-	THC induced triphasic BP response (↓ BP via vagal stimulation, then ↑ BP not dependent on sympathetic activity followed by ↓ BP due to central decrease in sympathetic tone).
Kawasaki 1980 [23]	Investigated the effect of THC on the CV system and behavior changes	Rats Anaesthetised (urethane, ip)	29	1–5 mg/kg	i.v.	Post-anaesthesia	Continues for 70 m post-drug	-	THC induced CV effects (↓ HR and ↑ BP) through vagal activity, and influence behavior changes to brain stimulation.
Schmeling 1981 [34]	Investigated the effect of THC on hypothalamus	Cats Anaesthetised (urethane, ip)	12	2 mg/kg	i.v.	Post-anaesthesia	Continues for 30 m post-drug	-	THC produced significant reductions in HR and BP and attenuated the pressor response threshold suggesting that THC reduces sympathetic activity.
Estrada 1987 [35]	Investigated the CV effects of THC	Rats Anaesthetised (pentobarbital, ip)	28	0.078–5 mg/kg	i.v.	Post-anaesthesia	3-12 min post-drug	-	THC produced adverse effects on the CV system (↓ HR and ↓ BP)
Krowicki 1999 [36]	Investigated whether CB_1_ activation by THC inhibits gastric motor function	Rats Anaesthetised (ketamine and xylazine)	36	0.02–2 mg/kg	i.v.	Post-anaesthesia	Continues for 10 m post-drug	-	THC decreased gastric motor function, HR, and BP via autonomic effects mediated by CB_1_.
**Conscious animals**									
Kaymakcalan 1974 [37]	Investigated chronic effects of THC on HR	Rats Conscious	20	10 mg/kg	s.c.	Single dose per day for 16 days	Hourly interval to 6 h on the 1st, 4th, 8th and 16th days	-	THC produced marked reduction in HR
Borgen 1974 [38]	Examined possible interaction of CBD on THC effects	Rabbits Conscious	8	3 mg/kg	i.v.	Pre-test	Pre-drug and hourly interval to 7 h post-drug	C: HR:264; T: HR:276	CBD reduced the hypothermic effect of THC and attenuated the depressant effects of THC on respiration, rectal temperature and HR
Brown 1974 [20]	Investigated CV response to THC	Bats Conscious	12	100 and 200 mg/kg	i.p.	Pre-test	Pre-drug and continues for 145 m post-drug	C: HR:436, BP:101; T: HR:390, BP:114	THC induced hypothermia and reduction in HR and BP.
Osgood 1977 [22]	Investigated THC effects on HR	Rats Conscious	18	0.5 mg/kg	i.p.	Pre-test	Continues for 30 m post-drug	-	THC had minimal effect on BP and caused an increase in HR, which may be related to central mediation release of epinephrine from adrenal gland.
Kawasaki 1980 [23]	Investigated the effects of THC on the CV system and behavior changes	Rats Conscious	21	4–8 mg/kg	i.p.	Pre-test	Continues for 2 h post-drug	-	THC induced CV effects (↓ HR and ↑ BP) through vagal activity, and influenced behavior changes to brain stimulation.
Matsuzaki 1987 [39]	Examined the effects of THC on EEG, body temperature, and HR	Monkeys Conscious	6	0.4–4 mg/kg	i.p.	Pre-test	Continues for 5 h post-drug	-	THC induced reduction in HR and hypothermia and induced responses of EGG along with behavioral depression and alertness.
Hayakawa 2007a [40]	Investigated CBD and THC effects on ischemic brain damage	Stroke Mice Conscious	17	10 mg/kg	i.p.	Pre-, 3 and 4 h post-occlusion, and 1 and 2 h post-reperfusion	BP and HR: pre-reperfusion. CBF: continued 4 h post-occlusion and 1 post-reperfusion	-	Pre and post-ischemic treatment with CBD induced neuroprotection, whereas only preischemic treatment with THC induced neuroprotection. THC increased CBF with no effects on BP or HR
Hayakawa 2007b [41]	Explored the development of tolerance of THC and CBD neuroprotection	Stroke Mice Conscious	7	10 mg/kg	i.p.	Pre-occlusion and 3 h post-occlusion. Single dose per day for 14 days	During 4 h and on day 14 post-occlusion	-	Repeated treatment with CBD, but not THC, induced neuroprotection with development of tolerance. THC increased CBF on day 1 only with no effects on BP or HR.
**Stress and hypertensive animal models**									
Williams 1973 [42]	Studied the effects of THC on BP	Rats Stress	30	20 mg/kg	s.c.	Single dose per day for 4 days	Pre-drug, 4 h, 48 and 96 h post-drug	C: BP:128; T: BP:129	THC reduced BP
Birmingham 1973 [43]	Studies the effects of THC on BP	Rats Hypertensive	10	3 mg/kg	i.p.	Single dose per day for 7 days	Hourly to 5 h for 7 days	-	THC reduced BP
Kosersky 1978 [44]	Examined the antihypertensive effects of THC	Rats Hypertensive	12	25 mg/kg	Oral	Single dose per day for 10 days	4 h and every day for 14 days post-drug	-	THC effectively reduced BP to the same degree over the treatment period.
**Humans**									
Karniol 1973 [45]	Compared the effects of 8-THC and 9-THC	Human Healthy	21	5–20 mg	Inhale	Pre-test	Avrg. of 20 m post-drug	C: HR:82; T: HR:85	9-THC was twice as active as 8-THC in increasing HR and caused more subjective symptoms.
Karniol 1975 [46]	Examined the interaction between THC and CBN	Human Healthy	5 (M)	25 mg	Oral	Pre-test	50, 70 and 160 m post-drug	-	THC induced increase in HR and psychological effects. No change on THC effects when combined with CBN
Zimmer 1976 [47]	Examined changes of somatic parameters post-THC	Human Healthy	36	250 µg/kg	Oral	Pre-test	Pre-drug and 4 h post-drug	C: HR:87.9, BP:127.5; T: HR:89, BP:123	THC raised HR with no changes on other parameters including BP
Haney 2007 [48]	Determined the effects of naltrexone in combination with THC	Human Healthy	21 (11 M & 10 F)	2.5–10 mg	Oral	Pre-test	Continues for 6 h post-drug	-	Naltrexone enhanced intoxication effects of THC; THC increased HR
Beaumont 2009 [21]	Evaluated whether THC has inhibitory effect on transient esophageal sphincter	Human Healthy	18 (M)	10 and 20 mg	Oral	Pre-test	Continues for 4 h post-drug	C: HR:59; T: HR:59	THC inhibited the increased induced meal transient esophageal sphincter relaxation. THC increased HR and decreased BP
Klooker 2011 [49]	Assessed the effect of THC on rectal sensation	Human Healthy and IBD	10 and 12	5 and 10 mg	Oral	Pre-test	Continues for 105 m post-drug	-	THC had no effect on rectal perception to distension. THC increased HR with no effect on BP

Abbreviations: BP: blood pressure, BF: Blood flow, C: control group, CB_1_: cannabinoid receptor 1, CBD: Cannabidiol, CBF: cerebral blood flow, CBN: cannabinol, CV: cardiovascular, D: THC treated group, F: females, G: gender, h: hour(s), HR: heart rate, , IBD: inflammatory bowel disease i.p.: intraperitoneal, i.v.: intravenous, M: males, m: minute(s), s.c.: Subcutaneous, T: treatment group, THC: ∆^9^-Tetrahydrocannabinol. ↑: increased, ↓: decreased. * Basal parameters values before intervention (i.e., anaesthetic agents or THC). The units of the parameters are HR: beats/m, BP: mmHg, BF: mL/m.
